# Isopropyl 2-(5-iodo-3-methyl­sulfinyl-1-benzofuran-2-yl)acetate

**DOI:** 10.1107/S1600536808037471

**Published:** 2008-11-20

**Authors:** Hong Dae Choi, Pil Ja Seo, Byeng Wha Son, Uk Lee

**Affiliations:** a Department of Chemistry, Dongeui University, San 24 Kaya-dong Busanjin-gu, Busan 614-714, Republic of Korea; b Department of Chemistry, Pukyong National University, 599-1 Daeyeon 3-dong Nam-gu, Busan 608-737, Republic of Korea

## Abstract

In the title compound, C_14_H_15_IO_4_S, the O atom and the methyl group of the methyl­sulfinyl substituent lie on opposite sides of the plane of the benzofuran fragment. The crystal structure is stabilized by C—H⋯π inter­actions between a methyl H atom and the benzene ring of an adjacent mol­ecule, and by weak inter­molecular C—H⋯O hydrogen bonds.

## Related literature

For the crystal structures of similar isopropyl 2-(3-methyl­sulfinyl-1-benzofuran-2-yl)acetate derivatives, see: Choi *et al.* (2008*a* ▶,*b* ▶).
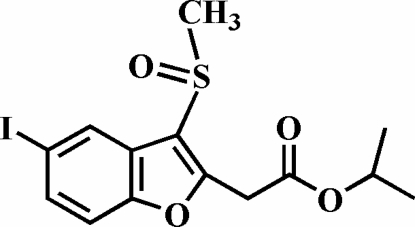


## Experimental

### 

#### Crystal data

C_14_H_15_IO_4_S
*M* *_r_* = 406.22
Triclinic, 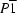

*a* = 8.0584 (7) Å
*b* = 10.1959 (9) Å
*c* = 10.8367 (9) Å
α = 70.369 (2)°
β = 81.926 (2)°
γ = 66.882 (1)°
*V* = 771.24 (12) Å^3^
*Z* = 2
Mo *K*α radiation
μ = 2.22 mm^−1^
*T* = 298 (2) K
0.30 × 0.20 × 0.10 mm

#### Data collection

Bruker SMART CCD diffractometer
Absorption correction: multi-scan (*SADABS*; Sheldrick, 1999 ▶) *T*_min_ = 0.595, *T*_max_ = 0.806
4396 measured reflections
2965 independent reflections
2639 reflections with *I* > 2σ(*I*)
*R*_int_ = 0.011

#### Refinement

*R*[*F* ^2^ > 2σ(*F* ^2^)] = 0.030
*wR*(*F* ^2^) = 0.079
*S* = 1.13
2965 reflections
182 parameters
H-atom parameters constrained
Δρ_max_ = 0.62 e Å^−3^
Δρ_min_ = −0.57 e Å^−3^

### 

Data collection: *SMART* (Bruker, 2001 ▶); cell refinement: *SAINT* (Bruker, 2001 ▶); data reduction: *SAINT*; program(s) used to solve structure: *SHELXS97* (Sheldrick, 2008 ▶); program(s) used to refine structure: *SHELXL97* (Sheldrick, 2008 ▶); molecular graphics: *ORTEP-3* (Farrugia, 1997 ▶) and *DIAMOND* (Brandenburg, 1998 ▶); software used to prepare material for publication: *SHELXL97*.

## Supplementary Material

Crystal structure: contains datablocks global, I. DOI: 10.1107/S1600536808037471/bq2107sup1.cif

Structure factors: contains datablocks I. DOI: 10.1107/S1600536808037471/bq2107Isup2.hkl

Additional supplementary materials:  crystallographic information; 3D view; checkCIF report

## Figures and Tables

**Table 1:** Hydrogen-bond geometry (Å, °) *Cg* is the centroid of the C2–C7 benzene ring.

*D*—H⋯*A*	*D*—H	H⋯*A*	*D*⋯*A*	*D*—H⋯*A*
C3—H3⋯O4^i^	0.93	2.57	3.451 (4)	159
C9—H9*B*⋯O4^ii^	0.97	2.41	3.373 (4)	170
C13—H13*C*⋯*Cg*^iii^	0.96	2.78	3.532 (5)	136

Symmetry codes: (i) 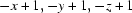; (ii) 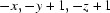; (iii) 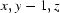.

**Fig. 1.**
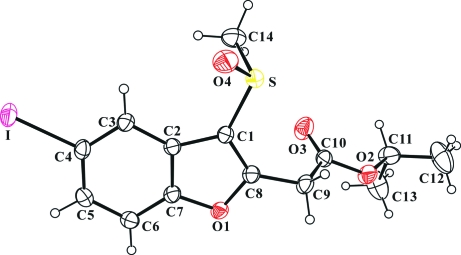
The molecular structure of the title compound, showing displacement ellipsoids drawn at the 30% probability level.

**Fig. 2.**
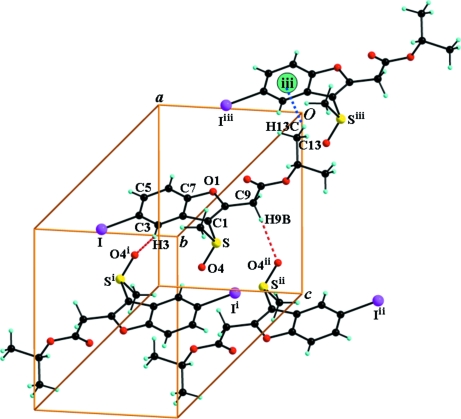
C—H···π and C—H···O interactions (dotted lines) in the title ompound. Cg denotes ring centroid. [Symmetry code: (i) -x+1, -y+1, -z+1; (ii) -x, -y+1, -z+1; (iii) x, y-1, z+1.]

## supplementary crystallographic information

### Comment

As a part of our ongoing studies of the synthesis and structure of isopropyl 2-(3-methylsulfinyl-1-benzofuran-2-yl)acetate analogues, we have recently described the crystal structures of isopropyl 2-(5-methyl-3-methylsulfinyl-1-benzofuran-2-yl)acetate (Choi *et al.*, 2008*a*) and isopropyl 2-(5-bromo-3-methylsulfinyl-1-benzofuran-2-yl) acetate (Choi *et al.*, 2008b). Here we report the crystal structure of the title compound, isopropyl 2-(5-iodo-3-methylsulfinyl-1-benzofuran-2-yl) acetate (Fig. 1). The benzofuran unit is essentially planar, with a mean deviation of 0.013 (2) Å from the least-squares plane defined by the nine constituent atoms. The molecular packing is stabilized by C—H···π interactions between a methyl H atom of isopropyl group and the benzene ring of the benzofuran unit, with a C13—H13C···Cg^iii^ separation of 2.78 Å (Fig. 2 and Table 1; Cg is the centroid of the C2–C7 benzene ring, symmetry code as in Fig. 2). Also weak intermolecular C—H···O hydrogen bonds in the structure were observed (Table 1 & Fig. 2).

### Experimental

77% 3-chloroperoxybenzoic acid (197 mg, 0.88 mmol) was added in small portions to a stirred solution of isopropyl 2-(5-iodo-3-methylsulfanyl-1-benzofuran-2-yl)acetate (321 mg, 0.8 mmol) in dichloromethane (30 ml) at 273 K. After being stirred for 3 h at room temperature, the mixture was washed with saturated sodium bicarbonate solution and the organic layer was separated, dried over magnesium sulfate, filtered and concentrated in vacuum. The residue was purified by column chromatography (hexane-ethyl acetate, 1:2 v/v) to afford the title compound as a colorless solid [yield 80%, m.p. 420–421 K; *R*_f_ = 0.63 (hexane-ethyl acetate, 1;2 v/v)]. Single crystals suitable for X–ray diffraction were prepared by evaporation of a solution of the title compound in acetone at room temperature. Spectroscopic analysis: ^1^H NMR (CDCl_3_, 400 MHz) δ 1.27 (d, J = 6.20 Hz, 6H), 3.07 (s, 3H), 4.0 (s, 2H), 5.01-5.07 (m, 1H), 7.29 (d, J = 8.80 Hz, 1H), 7.66 (d, J = 8.76 Hz, 1H), 8.29 (s, 1H); EI-MS 406 [M^+^].

### Refinement

All H atoms were geometrically positioned and refined using a riding model, with C—H = 0.93 Å for the aryl, 0.97 Å for the methylene, 0.98 Å for the methine, and 0.96 Å for the methyl H atoms. *U*_iso_(H) = 1.2Ueq(C) for the aryl, methine and methylene H atoms, and 1.5Ueq(C) for methyl H atoms.

### Crystal data

**Table:**

C_14_H_15_IO_4_S	*Z* = 2
*M**_r_* = 406.22	*F*_000_ = 400
Triclinic, *P*1	*D*_x_ = 1.749 Mg m^−^^3^
Hall symbol: -P 1	Melting point = 420–421 K
*a* = 8.0584 (7) Å	Mo *K*α radiation λ = 0.71073 Å
*b* = 10.1959 (9) Å	Cell parameters from 3094 reflections
*c* = 10.8367 (9) Å	θ = 2.5–28.2º
α = 70.369 (2)º	µ = 2.22 mm^−^^1^
β = 81.926 (2)º	*T* = 298 (2) K
γ = 66.882 (1)º	Block, colorless
*V* = 771.24 (12) Å^3^	0.30 × 0.20 × 0.10 mm

### Data collection

**Table:**

Bruker SMART CCD diffractometer	2965 independent reflections
Radiation source: fine-focus sealed tube	2639 reflections with *I* > 2σ(*I*)
Monochromator: graphite	*R*_int_ = 0.011
Detector resolution: 10.0 pixels mm^-1^	θ_max_ = 26.0º
*T* = 298(2) K	θ_min_ = 2.5º
φ and ω scans	*h* = −9→9
Absorption correction: multi-scan(SADABS; Sheldrick, 1999)	*k* = −12→10
*T*_min_ = 0.595, *T*_max_ = 0.806	*l* = −11→13
4396 measured reflections	

### Refinement

**Table:**

Refinement on *F*^2^	Secondary atom site location: difference Fourier map
Least-squares matrix: full	Hydrogen site location: inferred from neighbouring sites
*R*[*F*^2^ > 2σ(*F*^2^)] = 0.030	H-atom parameters constrained
*wR*(*F*^2^) = 0.079	*w* = 1/[σ^2^(*F*_o_^2^) + (0.0351*P*)^2^ + 0.6769*P*] where *P* = (*F*_o_^2^ + 2*F*_c_^2^)/3
*S* = 1.13	(Δ/σ)_max_ < 0.001
2965 reflections	Δρ_max_ = 0.62 e Å^−^^3^
182 parameters	Δρ_min_ = −0.57 e Å^−^^3^
Primary atom site location: structure-invariant direct methods	Extinction correction: none

### Special details

**Table:**

Geometry. All esds (except the esd in the dihedral angle between two l.s. planes) are estimated using the full covariance matrix. The cell esds are taken into account individually in the estimation of esds in distances, angles and torsion angles; correlations between esds in cell parameters are only used when they are defined by crystal symmetry. An approximate (isotropic) treatment of cell esds is used for estimating esds involving l.s. planes.
Refinement. Refinement of F^2^ against ALL reflections. The weighted R-factor wR and goodness of fit S are based on F^2^, conventional R-factors R are based on F, with F set to zero for negative F^2^. The threshold expression of F^2^ > 2sigma(F^2^) is used only for calculating R-factors(gt) etc. and is not relevant to the choice of reflections for refinement. R-factors based on F^2^ are statistically about twice as large as those based on F, and R- factors based on ALL data will be even larger.

### Fractional atomic coordinates and isotropic or equivalent isotropic displacement parameters (Å^2^)

**Table:**

	*x*	*y*	*z*	*U*_iso_*/*U*_eq_	
I	0.70596 (3)	0.79850 (3)	0.12855 (3)	0.05948 (12)	
S	0.25632 (12)	0.39419 (10)	0.46216 (8)	0.0450 (2)	
O1	0.1637 (3)	0.5266 (2)	0.0838 (2)	0.0396 (5)	
O2	−0.0175 (4)	0.1492 (3)	0.2759 (3)	0.0541 (6)	
O3	0.2647 (4)	0.1371 (3)	0.2867 (3)	0.0668 (8)	
O4	0.2538 (4)	0.5137 (3)	0.5137 (3)	0.0601 (7)	
C1	0.2512 (4)	0.4675 (3)	0.2898 (3)	0.0362 (6)	
C2	0.3469 (4)	0.5580 (3)	0.2042 (3)	0.0349 (6)	
C3	0.4715 (4)	0.6148 (4)	0.2202 (3)	0.0398 (7)	
H3	0.5164	0.5932	0.3022	0.048*	
C4	0.5252 (4)	0.7044 (3)	0.1086 (3)	0.0406 (7)	
C5	0.4622 (5)	0.7382 (4)	−0.0162 (3)	0.0430 (7)	
H5	0.5030	0.7988	−0.0884	0.052*	
C6	0.3394 (5)	0.6817 (4)	−0.0328 (3)	0.0413 (7)	
H6	0.2955	0.7026	−0.1149	0.050*	
C7	0.2851 (4)	0.5925 (3)	0.0795 (3)	0.0369 (7)	
C8	0.1443 (4)	0.4531 (3)	0.2138 (3)	0.0377 (7)	
C9	0.0157 (5)	0.3741 (4)	0.2441 (4)	0.0434 (7)	
H9A	−0.0662	0.4154	0.1712	0.052*	
H9B	−0.0556	0.3928	0.3207	0.052*	
C10	0.1072 (5)	0.2070 (4)	0.2695 (3)	0.0414 (7)	
C11	0.0424 (6)	−0.0125 (4)	0.2990 (4)	0.0661 (12)	
H11	0.1531	−0.0670	0.3506	0.079*	
C12	−0.1125 (10)	−0.0567 (7)	0.3724 (6)	0.104 (2)	
H12A	−0.2185	−0.0037	0.3191	0.125*	
H12B	−0.1363	−0.0315	0.4526	0.125*	
H12C	−0.0805	−0.1626	0.3919	0.125*	
C13	0.0701 (9)	−0.0397 (5)	0.1698 (6)	0.0926 (18)	
H13A	0.1579	−0.0006	0.1195	0.111*	
H13B	−0.0420	0.0096	0.1235	0.111*	
H13C	0.1124	−0.1454	0.1828	0.111*	
C14	0.4840 (6)	0.2608 (5)	0.4771 (4)	0.0647 (11)	
H14A	0.5657	0.3127	0.4443	0.097*	
H14B	0.4989	0.1947	0.4273	0.097*	
H14C	0.5092	0.2035	0.5675	0.097*	

### Atomic displacement parameters (Å^2^)

**Table:**

	*U*^11^	*U*^22^	*U*^33^	*U*^12^	*U*^13^	*U*^23^
I	0.05565 (17)	0.05111 (16)	0.0792 (2)	−0.03419 (12)	−0.00801 (12)	−0.00920 (12)
S	0.0496 (5)	0.0512 (5)	0.0385 (4)	−0.0270 (4)	−0.0031 (3)	−0.0082 (4)
O1	0.0425 (12)	0.0397 (12)	0.0418 (12)	−0.0179 (10)	−0.0069 (9)	−0.0131 (10)
O2	0.0601 (15)	0.0406 (13)	0.0698 (17)	−0.0273 (12)	−0.0179 (12)	−0.0099 (12)
O3	0.0509 (16)	0.0473 (15)	0.100 (2)	−0.0204 (13)	−0.0092 (15)	−0.0144 (15)
O4	0.0719 (18)	0.0681 (18)	0.0502 (15)	−0.0283 (15)	−0.0032 (13)	−0.0264 (13)
C1	0.0400 (16)	0.0352 (15)	0.0373 (17)	−0.0179 (13)	−0.0038 (13)	−0.0099 (13)
C2	0.0376 (15)	0.0296 (14)	0.0383 (17)	−0.0123 (12)	−0.0044 (12)	−0.0100 (12)
C3	0.0412 (17)	0.0383 (17)	0.0438 (18)	−0.0179 (14)	−0.0052 (13)	−0.0120 (14)
C4	0.0376 (16)	0.0324 (16)	0.055 (2)	−0.0157 (13)	−0.0010 (14)	−0.0135 (14)
C5	0.0472 (18)	0.0347 (16)	0.0434 (19)	−0.0159 (14)	0.0017 (14)	−0.0073 (14)
C6	0.0462 (18)	0.0385 (17)	0.0371 (17)	−0.0140 (14)	−0.0040 (13)	−0.0097 (13)
C7	0.0394 (16)	0.0315 (15)	0.0435 (18)	−0.0137 (13)	−0.0045 (13)	−0.0138 (13)
C8	0.0419 (16)	0.0336 (15)	0.0414 (17)	−0.0155 (13)	−0.0040 (13)	−0.0128 (13)
C9	0.0439 (18)	0.0441 (18)	0.0512 (19)	−0.0228 (15)	−0.0039 (14)	−0.0165 (15)
C10	0.050 (2)	0.0435 (18)	0.0382 (17)	−0.0264 (16)	−0.0039 (14)	−0.0100 (14)
C11	0.085 (3)	0.0381 (19)	0.078 (3)	−0.031 (2)	−0.034 (2)	0.0010 (19)
C12	0.178 (7)	0.097 (4)	0.080 (4)	−0.099 (5)	0.029 (4)	−0.032 (3)
C13	0.116 (4)	0.052 (3)	0.104 (4)	−0.032 (3)	0.044 (3)	−0.034 (3)
C14	0.061 (2)	0.059 (2)	0.060 (2)	−0.012 (2)	−0.0181 (19)	−0.006 (2)

### Geometric parameters (Å, °)

**Table:**

I—C4	2.101 (3)	C6—C7	1.385 (5)
S—O4	1.494 (3)	C6—H6	0.9300
S—C1	1.763 (3)	C8—C9	1.488 (4)
S—C14	1.794 (4)	C9—C10	1.509 (5)
O1—C7	1.375 (4)	C9—H9A	0.9700
O1—C8	1.375 (4)	C9—H9B	0.9700
O2—C10	1.335 (4)	C11—C13	1.487 (7)
O2—C11	1.465 (4)	C11—C12	1.521 (7)
O3—C10	1.192 (4)	C11—H11	0.9800
C1—C8	1.350 (4)	C12—H12A	0.9600
C1—C2	1.445 (4)	C12—H12B	0.9600
C2—C7	1.390 (4)	C12—H12C	0.9600
C2—C3	1.395 (4)	C13—H13A	0.9600
C3—C4	1.380 (5)	C13—H13B	0.9600
C3—H3	0.9300	C13—H13C	0.9600
C4—C5	1.396 (5)	C14—H14A	0.9600
C5—C6	1.382 (5)	C14—H14B	0.9600
C5—H5	0.9300	C14—H14C	0.9600
			
O4—S—C1	107.22 (15)	C10—C9—H9A	108.9
O4—S—C14	106.49 (19)	C8—C9—H9B	108.9
C1—S—C14	97.67 (18)	C10—C9—H9B	108.9
C7—O1—C8	106.2 (2)	H9A—C9—H9B	107.7
C10—O2—C11	118.3 (3)	O3—C10—O2	125.4 (3)
C8—C1—C2	107.2 (3)	O3—C10—C9	125.4 (3)
C8—C1—S	124.0 (2)	O2—C10—C9	109.1 (3)
C2—C1—S	128.7 (2)	O2—C11—C13	108.2 (3)
C7—C2—C3	119.4 (3)	O2—C11—C12	105.4 (4)
C7—C2—C1	104.7 (3)	C13—C11—C12	109.8 (4)
C3—C2—C1	135.9 (3)	O2—C11—H11	111.1
C4—C3—C2	116.9 (3)	C13—C11—H11	111.1
C4—C3—H3	121.6	C12—C11—H11	111.1
C2—C3—H3	121.6	C11—C12—H12A	109.5
C3—C4—C5	123.2 (3)	C11—C12—H12B	109.5
C3—C4—I	118.3 (2)	H12A—C12—H12B	109.5
C5—C4—I	118.5 (2)	C11—C12—H12C	109.5
C6—C5—C4	120.2 (3)	H12A—C12—H12C	109.5
C6—C5—H5	119.9	H12B—C12—H12C	109.5
C4—C5—H5	119.9	C11—C13—H13A	109.5
C5—C6—C7	116.4 (3)	C11—C13—H13B	109.5
C5—C6—H6	121.8	H13A—C13—H13B	109.5
C7—C6—H6	121.8	C11—C13—H13C	109.5
O1—C7—C6	125.3 (3)	H13A—C13—H13C	109.5
O1—C7—C2	110.7 (3)	H13B—C13—H13C	109.5
C6—C7—C2	123.9 (3)	S—C14—H14A	109.5
C1—C8—O1	111.1 (3)	S—C14—H14B	109.5
C1—C8—C9	132.6 (3)	H14A—C14—H14B	109.5
O1—C8—C9	116.3 (3)	S—C14—H14C	109.5
C8—C9—C10	113.4 (3)	H14A—C14—H14C	109.5
C8—C9—H9A	108.9	H14B—C14—H14C	109.5
			
O4—S—C1—C8	−135.0 (3)	C3—C2—C7—O1	180.0 (3)
C14—S—C1—C8	115.0 (3)	C1—C2—C7—O1	−1.5 (3)
O4—S—C1—C2	41.4 (3)	C3—C2—C7—C6	−0.2 (5)
C14—S—C1—C2	−68.6 (3)	C1—C2—C7—C6	178.3 (3)
C8—C1—C2—C7	0.6 (3)	C2—C1—C8—O1	0.6 (4)
S—C1—C2—C7	−176.3 (2)	S—C1—C8—O1	177.6 (2)
C8—C1—C2—C3	178.7 (4)	C2—C1—C8—C9	179.9 (3)
S—C1—C2—C3	1.9 (6)	S—C1—C8—C9	−3.1 (5)
C7—C2—C3—C4	0.5 (4)	C7—O1—C8—C1	−1.5 (3)
C1—C2—C3—C4	−177.4 (3)	C7—O1—C8—C9	179.1 (3)
C2—C3—C4—C5	−0.6 (5)	C1—C8—C9—C10	−78.0 (5)
C2—C3—C4—I	177.9 (2)	O1—C8—C9—C10	101.3 (3)
C3—C4—C5—C6	0.3 (5)	C11—O2—C10—O3	−3.5 (5)
I—C4—C5—C6	−178.2 (2)	C11—O2—C10—C9	179.5 (3)
C4—C5—C6—C7	0.1 (5)	C8—C9—C10—O3	12.3 (5)
C8—O1—C7—C6	−177.9 (3)	C8—C9—C10—O2	−170.7 (3)
C8—O1—C7—C2	1.8 (3)	C10—O2—C11—C13	−92.6 (4)
C5—C6—C7—O1	179.7 (3)	C10—O2—C11—C12	150.0 (4)
C5—C6—C7—C2	−0.1 (5)		

### Hydrogen-bond geometry (Å, °)

**Table:**

*D*—H···*A*	*D*—H	H···*A*	*D*···*A*	*D*—H···*A*
C3—H3···O4^i^	0.93	2.57	3.451 (4)	159
C9—H9B···O4^ii^	0.97	2.41	3.373 (4)	170
C13—H13C···Cg^iii^	0.96	2.78	3.532 (5)	136

Symmetry codes: (i) −*x*+1, −*y*+1, −*z*+1; (ii) −*x*, −*y*+1, −*z*+1; (iii) *x*, *y*−1, *z*.
